# Supplementary material for: Towards a fully automated surveillance of well-being status in laboratory mice using deep learning: Starting with facial expression analysis
Source: PLoS One. 2020 Apr 15;15(4):e0228059. doi: 10.1371/journal.pone.0228059 (PMC7159220; doi:10.1371/journal.pone.0228059)
Supplement: S1 File — (PDF) [file pone.0228059.s001.pdf]

1 Number of action units visible

The following table shows how the face detector performs, when not all facial action units are visible. The numbers are the result of manually evaluating this on a random sample of 1000 images from the original dataset. The first column contains the number of facial action units used in the MGS, that are visible on the image. The second column gives the ratio of images, on which exactly this number of action units can be seen. The third column contains the detection rate on that corresponding subset of images - i.e. on what proportion of images with a specific number of action units on them our final face detection procedure successfully found a face.

S1 Table1

Number of action units visible	frequency of occurrence in the sample	detection rate of our final face detector
0	0.004	0.000
1	0.005	0.800
2	0.028	0.214
3	0.028	0.464
4	0.193	0.648
5	0.742	0.729

2 Sex differences

We created Fig 5 and Fig 6 also separated by sex. Results can be seen in the following figures.

2.1 Isoflurane anesthesia

See Figures 1 and 2. MGS data represent mean MGS scores averaged over four human scorers. The box represents the interquartile range (IQR), box edges are the 25th and 75th percentile. The whiskers represent values which are no greater than 1.5 × IQR. Outliers were excluded from the figure. These figures contain data from Hohlbaum et al. [1].

2.2 Ketamine/xylazine anesthesia

See Figures 3 and 4. MGS data represent mean MGS scores averaged over four human scorers. The box represents the interquartile range (IQR), box edges are the 25th and 75th percentile. The whiskers represent values which are no greater than 1.5 × IQR. Outliers were excluded from the figure. These figures contain data from Hohlbaum et al. [2].

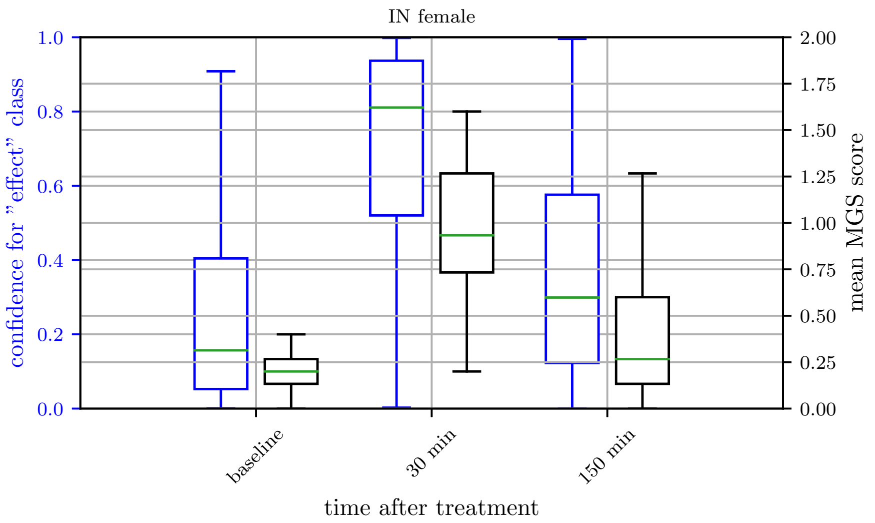

Figure 1: Network confidence over time for female mice and isoflurane anesthesia. Box plots of human labeled Mouse Grimace Scale (MGS) score (grey) and confidence for "post-anesthetic/surgical effect" class of ResNet architecture (blue) for isoflurane anesthesia (IN). Scores were obtained from 33 female C57BL/6JRj mice.

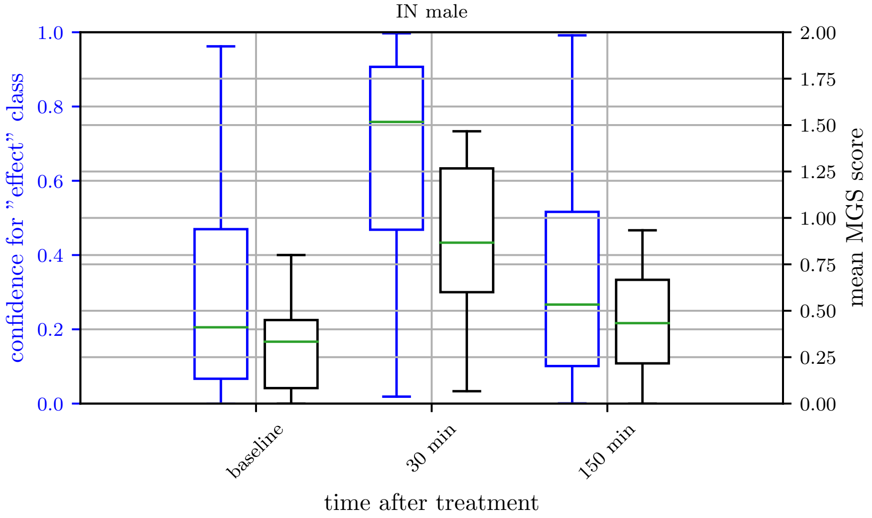

Figure 2: Network confidence over time for male mice and isoflurane anesthesia. Box plots of human labeled Mouse Grimace Scale (MGS) score (grey) and confidence for "post-anesthetic/surgical effect" class of ResNet architecture (blue) for isoflurane anesthesia (IN). Scores were obtained from 32 male C57BL/6JRj mice.

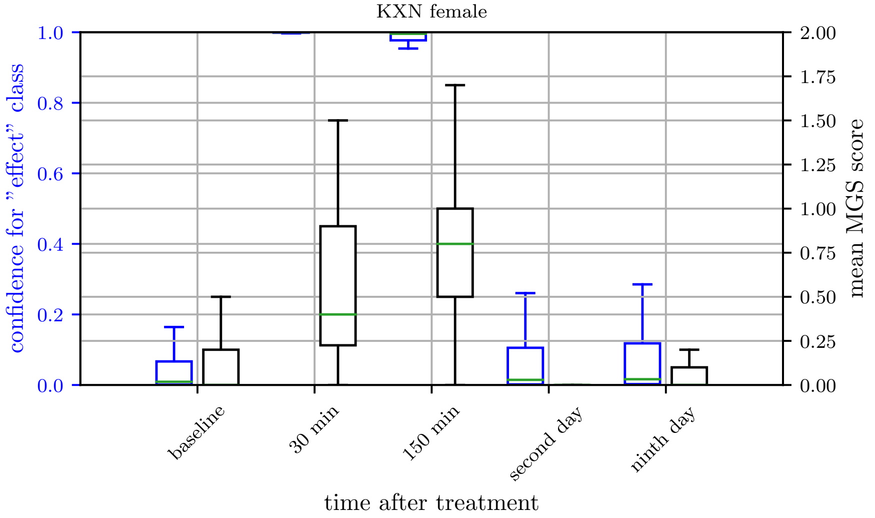

Figure 3: Network confidence over time for female mice and ketamine/xylazine anesthesia. Box plots of human labeled Mouse Grimace Scale (MGS) score (grey) and confidence for "post-anesthetic/surgical effect" class of ResNet architecture (blue) for ketamine/xylazine anesthesia (IN). Scores were obtained from 28 female C57BL/6JRj mice.

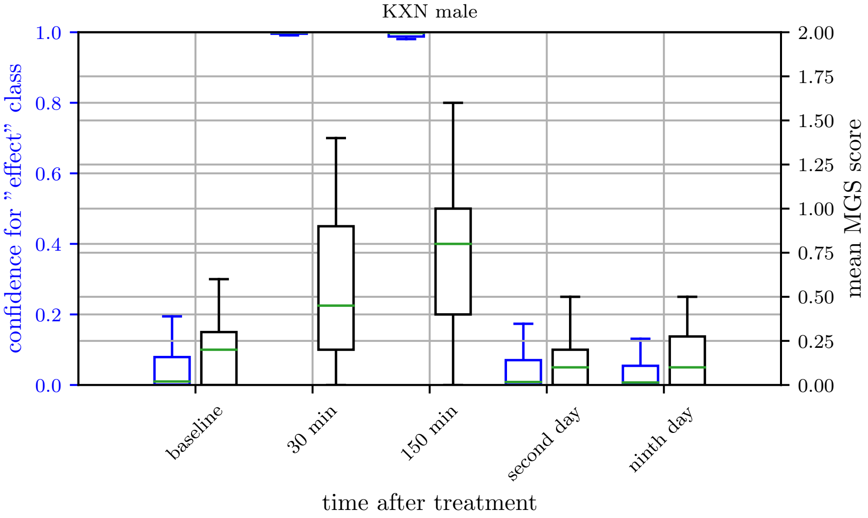

Figure 4: Network confidence over time for male mice and ketamine/xylazine anesthesia. Box plots of human labeled Mouse Grimace Scale (MGS) score (grey) and confidence for "post-anesthetic/surgical effect" class of ResNet architecture (blue) for ketamine/xylazine anesthesia (IN). Scores were obtained from 30 male C57BL/6JRj mice.

References

1. Hohlbaum K, Bert B, Dietze S, Palme R, Fink H, Thöne-Reineke C. Severity classification of repeated isoflurane anesthesia in C57BL/6JRj mice—Assessing the degree of distress. PloS One. 2017;12:e0179588.

2. Hohlbaum K, Bert B, Dietze S, Palme R, Fink H, Thöne-Reineke C. Impact of repeated anesthesia with ketamine and xylazine on the well-being of C57BL/6JRj mice. PloS One. 2018;13:e0203559.
