# Supplementary figures and images for: Towards a fully automated surveillance of well-being status in laboratory mice using deep learning: Starting with facial expression analysis
Source: PLoS One. 2020 Apr 15;15(4):e0228059. doi: 10.1371/journal.pone.0228059 (PMC7159220; doi:10.1371/journal.pone.0228059)

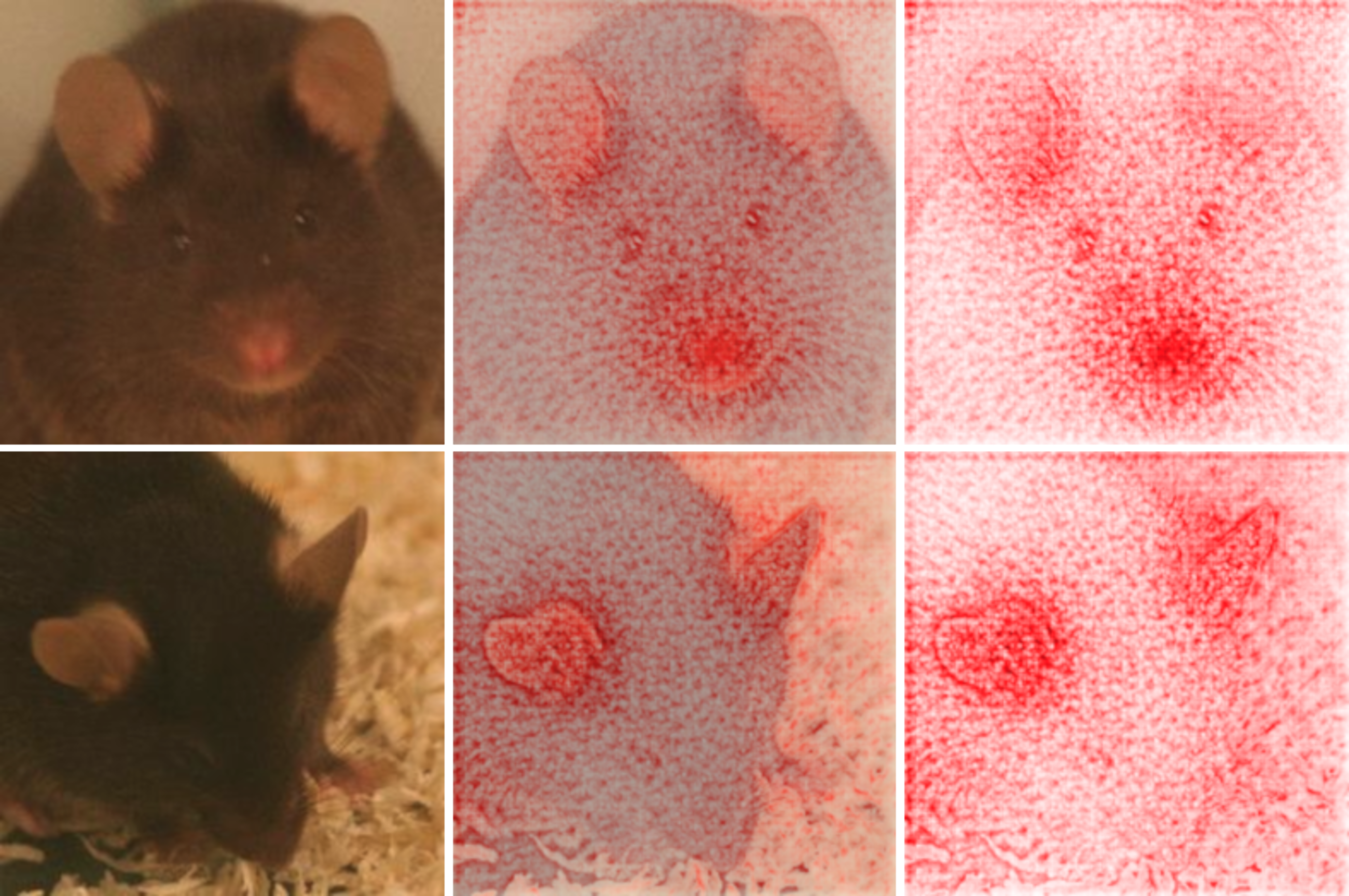

Supplement: S1 Fig — (TIF) [file pone.0228059.s002.tif]

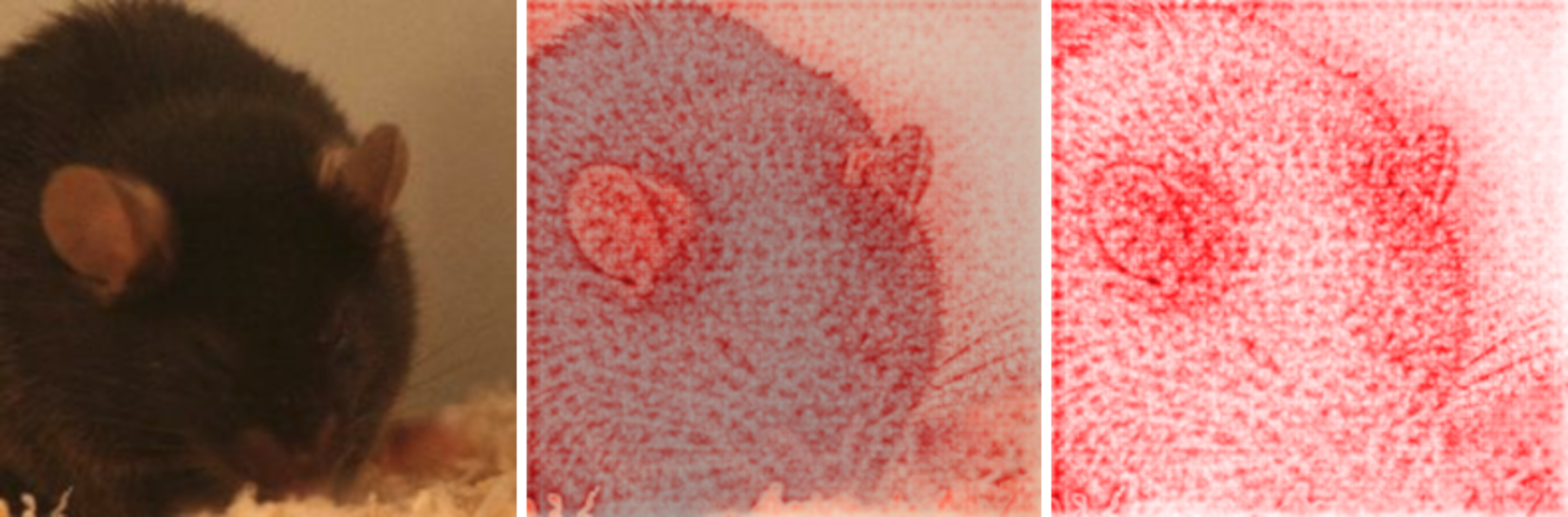

Supplement: S2 Fig — (TIF) [file pone.0228059.s003.tif]
